# Nutrient Media in the Local Treatment of Ulcers

**Published:** 1909-07-10

**Authors:** 


### NUTRIENT MEDIA IN THE LOCAL TREATMENT OF ULCERS.

There are many ulcers that get well easily enough under the simplest treatment; there are others, on the other hand, that stubbornly resist treatment altogether. It is by no means a new suggestion that such stubborn ulcers should be treated with nutrient media, but it is a line of treatment that is apt to be forgotten by the practitioner. It has a strong advocate in Professor Carter S. Cole in New York.

Anything that will furnish nutrition to tissues will also serve as a culture medium for bacteria, of course, and therefore a good deal of careful supervision is required in its use; but there is no reason why the nutrient application should not be used in conjunction with an antiseptic which will obviate the risks of further sepsis. Bed-sores, for instance, are amongst the most difficult of things to cure; they may need to be fomented with boric acid four-hourly to keep them clean, and yet the granulation tissue in their bases is slow in growing. If dry and sterile powdered peptone is sprinkled over the surface of such an ulcer each time it is dressed it is wonderful how much more quickly the granulations will grow than before. A slow, indolent ulcer may be converted into one that is granulating healthily and vigorously in quite a short time.

Amongst the different nutrient media that may be applied to ulcers in this way one composed of alcohol and water, ether-extracted fat, gelatine and albumen, peptone, creatine and meat extractives, and salts proportionate to those in ordinary tissues, serves well. It is simpler, however, to use either cod liver oil, or powdered peptone, or a combination of these two.

If there is no sepsis that requires treatment by fomentations at the same time, a piece of sterile gauze saturated with the above nutrient solution may be laid over the ulcer; over this a piece of gutta percha tissue or oiled silk extending beyond the edges of the gauze in order to retain heat and prevent contamination, and outside this a piece of dry gauze and a good bandage smoothly applied. This dressing should be changed daily, or certainly at no longer interval than forty-eight hours. When changing the dressing the wound surface and its surroundings are washed clean with dilute alcohol, and the process is then repeated as before. The improvement is almost immediate. The gauze becomes white where it lies in contact with the ulcer, and it is very little changed where it has been in contact with sound skin.

One important point claimed by Professor Cole in favour of nutrient dressings is that after deep burns the repair is so much more rapid that there is far less scarring than there otherwise would almost certainly be.

